# Supplementary material for: Content-rich biological network constructed by mining PubMed abstracts
Source: BMC Bioinformatics. 2004 Oct 8;5:147. doi: 10.1186/1471-2105-5-147 (PMC528731; doi:10.1186/1471-2105-5-147)
Supplement: Additional File 2 — The original results of the above study (non-essential files are deleted to keep the file size under the limit set by BMC bioinformatics). [file 1471-2105-5-147-S2.bz2 › chilibotAdditionalFile2/dip40/385ID9252374E9850/html/THPO_MPL.html]

 


 **THPO** and **MPL** 
  
Found 539 abstracts in PubMed, retrieved 40.  
 

 What does Google say? 
 PDF only 
| .edu only 

---

**Interactive relationship** (e.g. stimulation, inhibition, etc)

**Inhibitory relationship**- IgG fractions purified from anti c  **Mpl**   [ **MPL** ]  antibody positive sera bound to c  **Mpl**   [ **MPL** ]  expressed on the cell surface and inhibited TPO  [ **THPO** ]  dependent cell proliferation and megakaryocyte colony formation.  Ref: 12209520 Arthritis Rheum, 2002
- It is likely that the impaired thrombopoiesis in these patients is mediated by the anti c  **Mpl**   [ **MPL** ]  antibody, which functionally blocks an interaction between TPO  [ **THPO** ]  and c  **Mpl**   [ **MPL** ] .  Ref: 12209520 Arthritis Rheum, 2002
- Our case report suggests that AMT in patients with SSc could be mediated by the anti c  **Mpl**   [ **MPL** ]  antibody, which functionally blocks an interaction between thrombopoietin  [ **THPO** ]  and c  **Mpl**   [ **MPL** ] .  Ref: 12794833 Arthritis Rheum, 2003
**Neutral relationship**- One mutant  **Mpl**  type SAG Delta GCRMplTmR successfully lost the responsiveness to TPO  [ **THPO** ]  without affecting the Tm dependence.  Ref: 12646182 Biochem Biophys Res Commun, 2003
- The cloning of thrombopoietin  [ **THPO** ]  TPO  [ **THPO** ] , followed by the analysis of TPO  [ **THPO** ]  and c  **mpl**   [ **MPL** ]  its cellular receptor knockout mice confirmed its role as the primary regulator of thrombopoiesis.  Ref: 12650174 J Gravit Physiol, 2001
- Thrombopoietin  [ **THPO** ]  TPO  [ **THPO** ]  is a hematopoietic cytokine that regulates megakaryocytosis and thrombocytosis by binding to its receptor c  **Mpl**   [ **MPL** ] .  Ref: 12841634 Mol Cell Biochem, 2003
- Thrombopoietin  [ **THPO** ]  TPO  [ **THPO** ]  is the major cytokine which is involved in platelet production and exerts its effects via the receptor c  **MPL**   [ **MPL** ] .  Ref: 12167989 Sheng Wu Hua Xue Yu Sheng Wu Wu Li Xue Bao Shanghai < MedlineTA>Sheng Wu Hua Xue Yu Sheng Wu Wu Li Xue Bao Shanghai , 2003
- In several laboratories TPO  [ **THPO** ]  was cloned based on it s binding to the product of the proto oncogene c  **mpl**   [ **MPL** ] , at the time an orphan cytokine receptor, and was subsequently found to affect nearly all aspects of thrombopoiesis.  Ref: 12430879 Int J Hematol, 2002
- Interestingly, decreased c  **MPL**   [ **MPL** ]  and elevated PRV 1 were also observed in patients with hereditary thrombocythemia HT who carry a mutation in the thrombopoietin  [ **THPO** ]  TPO  [ **THPO** ]  gene.  Ref: 12730106 Blood< MedlineTA>Blood, 2002
- Although it is clear that thrombopoietin  [ **THPO** ]  is the primary regulator of thrombopoiesis, several lines of evidence indicate that the hormone affects multiple aspects of hematopoiesis the in vivo administration of TPO  [ **THPO** ]  increases marrow levels of erythroid, myeloid, and megakaryocytic progenitor cells and its genetic elimination or that of its receptor c  **mpl**   [ **MPL** ]  reduces the numbers of these cells.  Ref: 12799280 Ann N Y Acad Sci< MedlineTA>Ann N Y Acad Sci, 2002

**Non-interactive relationship** (e.g. studied together, co-existance, homology, etc.)

- In familial disorders, pathological mutations have been identified in some, but by no means all, cases EPO receptor mutations in PFCP, TPO  [ **THPO** ]  mutations in FT and, conversely, TPO  [ **THPO** ]  receptor c  **mpl**   [ **MPL** ]  mutations in CAMT.  Ref: 12430942 Int J Hematol, 2002
- These agents include recombinant human interleukin 11, recombinant human thrombopoietin  [ **THPO** ] , and c  **Mpl**   [ **MPL** ]  ligand  [ **THPO** ]  mimetics.  Ref: 12469931 Oncology Huntingt , 2002
- PLZF overexpression upmodulates the megakaryocytic specific markers CD42a, CD42b, CD61, PF4 and induces the thrombopoietin  [ **THPO** ]  receptor TpoR  [ **MPL** ] .  Ref: 12242665 Oncogene, 2002
- Megakaryocytes, the platelet precursors, are induced to differentiate in response to  **Mpl**  ligand  [ **THPO** ] .  Ref: 12220674 FEBS Lett, 2002
- Since the purification of TPO  [ **THPO** ]  in 199 recombinant forms of the c  **Mpl**   [ **MPL** ]  ligand  [ **THPO** ]  recombinant human thrombopoietin  [ **THPO** ]  rhTPO and pegylated recombinant human megakaryocyte growth and development factor  [ **THPO** ]  PEG rHuMGDF have undergone extensive clinical investigation.  Ref: 12411315 Blood, 2002
- Thrombopoietin  [ **THPO** ]  TPO  [ **THPO** ] , the c  **Mpl**   [ **MPL** ]  ligand  [ **THPO** ] , is the primary physiologic regulator of megakaryocyte and platelet development.  Ref: 12411315 Blood, 2002
- we show a similar modulation by TGF beta1 of a fourth receptor the TPO  [ **THPO** ]  receptor  **MPL** .  Ref: 12725536 Oncol Res, 2003
- These data help identify the receptor motifs involved in TPO  [ **THPO** ]  induced internalization of  **Mpl**  and suggest that  **Mpl**  translocation may be necessary for normal cellular proliferation.  Ref: 12623841 Blood< MedlineTA>Blood, 2003
- Anti c  **Mpl**   [ **MPL** ]  thrombopoietin  [ **THPO** ]  receptor autoantibody induced amegakaryocytic thrombocytopenia in a patient with systemic sclerosis.  Ref: 12794833 Arthritis Rheum, 2003
- Because thrombopoietin  [ **THPO** ]  TPO  [ **THPO** ]  and its receptor, c  **Mpl**   [ **MPL** ] , regulate primitive hematopoietic populations, including bone marrow hematopoietic stem cells, we investigated whether TPO  [ **THPO** ]  acts on the hemangioblasts that derive from differentiation of embryonic stem cells in vitro.  Ref: 12743322 Stem Cells, 2003
- OBJECTIVES We sought to investigate potential mechanisms of increased serum thrombopoietin  [ **THPO** ]  TPO  [ **THPO** ]  concentrations in myelofibrosis with myeloid metaplasia MMM by simultaneously measuring serum TPO  [ **THPO** ] , platelet and megakaryocyte MK numbers, and MK c  **Mpl**   [ **MPL** ]  expression.  Ref: 12068799 Eur J Haematol, 2002
- We aimed to evaluate the proliferative signal transduction events following the activation of c  **mpl**   [ **MPL** ]  and we stimulated B1647 cells with TPO  [ **THPO** ]  40 ng mL for, 15 and 30 minutes.  Ref: 12495897 Haematologica, 2002
- In addition, we constructed various mutant  **Mpl**  type SAGs to abolish the responsiveness to endogenous TPO  [ **THPO** ]  while retaining Tm dependency.  Ref: 12646182 Biochem Biophys Res Commun, 2003
- Here we report that stability of the megakaryocyte specific platelet factor 4 PF4 mRNA is substantially augmented in the presence of  **Mpl**  ligand  [ **THPO** ] .  Ref: 12220674 FEBS Lett, 2002
- Clinical findings, autoantibody profiles, and serum TPO  [ **THPO** ]  levels were compared between SLE patients with and without anti c  **Mpl**   [ **MPL** ]  antibodies.  Ref: 12209520 Arthritis Rheum, 2002
- Autoantibody to c  **Mpl**   [ **MPL** ]  thrombopoietin  [ **THPO** ]  receptor in systemic lupus erythematosus relationship to thrombocytopenia with megakaryocytic hypoplasia.  Ref: 12209520 Arthritis Rheum, 2002
- Like CAMT patients, mice lacking the TPO  [ **THPO** ]  receptor c  **Mpl**   [ **MPL** ]  demonstrate a major reduction of early hematopoietic progenitor cells of all lineages.  Ref: 12799278 Ann N Y Acad Sci, 2003
- Thrombopoietin  [ **THPO** ]  Tpo  [ **THPO** ]  and its receptor, c  **mpl**   [ **MPL** ] , are expressed in murine embryonic stem ES cells.  Ref: 12372629 FEBS Lett, 2002
- However, serum TPO  [ **THPO** ]  did not correlate with either bone marrow MK number or c  **Mpl**   [ **MPL** ]  expression.  Ref: 12068799 Eur J Haematol, 2002
- Suitable consensus sequences were combined in one molecule, resulting in glycosylation analogs of rHuEPO, leptin, and  **Mpl**  ligand  [ **THPO** ] .  Ref: 12612588 Nat Biotechnol, 2003
- Two alternative hypotheses for the mechanism of regulation have been proposed 1 platelet and or megakaryocyte mass regulate circulating TPO  [ **THPO** ]  levels by binding to TPO  [ **THPO** ]  through TPO  [ **THPO** ]  receptors c  **MPL**   [ **MPL** ] , with subsequent internalization and degradation of the protein.  Ref: 12479847 Leuk Res, 2003
- A pathogenic role for the anti c  **Mpl**   [ **MPL** ]  antibody was evaluated by examining its inhibitory effect on TPO  [ **THPO** ]  dependent cell proliferation and megakaryocyte colony formation.  Ref: 12209520 Arthritis Rheum, 2002
- We examined whole embryos or the cells isolated from embryo proper and yolk sacs and identified both Tpo  [ **THPO** ]  and c  **mpl**   [ **MPL** ]  Tpo  [ **THPO** ]  receptor mRNA transcripts in tissues as early as embryonic day 6.5 E6.5.  Ref: 12393382 Blood, 2003
- Because these proteins were of three different classes rHuEPO is an N linked glycoprotein,  **Mpl**  ligand  [ **THPO** ]  an O linked glycoprotein, and leptin contains no carbohydrate, glycoengineering may be generally applicable as a strategy for increasing the in vivo activity and duration of action of proteins.  Ref: 12612588 Nat Biotechnol, 2003
- Aggregation of the Intracellular Domain of the Human Thrombopoietin  [ **THPO** ]  Receptor c  **MPL**   [ **MPL** ] .  Ref: 12167989 Sheng Wu Hua Xue Yu Sheng Wu Wu Li Xue Bao Shanghai < MedlineTA>Sheng Wu Hua Xue Yu Sheng Wu Wu Li Xue Bao Shanghai , 2003
- Our study is the first to suggest that  **Mpl**  ligand  [ **THPO** ]  induced lineage specification is also determined by message stabilization.  Ref: 12220674 FEBS Lett, 2002
- Her serum was strongly positive for anti c  **Mpl**   [ **MPL** ]  antibody, and IgG fraction purified from her serum inhibited thrombopoietin  [ **THPO** ]  dependent cell proliferation in vitro.  Ref: 12794833 Arthritis Rheum, 2003
- METHODS Megakaryocyte progenitor cell subpopulations from cord blood CB and mobilized peripheral blood PBSC were expanded in vitro in the presence of  **mpl**  ligand  [ **THPO** ] .  Ref: 12691920 Exp Hematol, 2003
- Expression in E6.5 yolk sac is compatible with a function of c  **mpl**   [ **MPL** ]  and its ligand, thrombopoietin  [ **THPO** ] , in the earliest stages of embryonic hematopoiesis.  Ref: 12130527 Blood, 2002
- To avoid the inadvertent effect of thrombopoietin  [ **THPO** ] , we used a chimeric receptor, PM R, composed of the extracellular domain of the prolactin receptor PRL R and the transmembrane and cytoplasmic domains of  **Mpl** .  Ref: 12200367 Blood, 2002
- Furthermore, TPO  [ **THPO** ]  stimulated cellular proliferation appears to be directly correlated with receptor internalization, indicating that internalization of the TPO  [ **THPO** ]   **Mpl**  complex may be essential for normal signal transduction.  Ref: 12623841 Blood< MedlineTA>Blood, 2002
- New insights are emerging into the role of the megakaryocytopoiesis regulator thrombopoietin  [ **THPO** ]  TPO  [ **THPO** ]  and its receptor, c  **Mpl**   [ **MPL** ] , in ET and related disorders, but TPO  [ **THPO** ]   **Mpl**  dynamics appear to be complex.  Ref: 12187022 Acta Haematol, 2002
- Thus, TPO  [ **THPO** ]  and  **Mpl**  may prove to be effective targets for both diagnosis and therapeutic management of MPD.  Ref: 12682875 Semin Hematol, 2003
- Recognition of defective platelet thrombopoietin  [ **THPO** ]  receptor  **Mpl**  expression and overexpression of the PRV 1 gene in PV neutrophils has led to studies demonstrating the potential use of these molecular abnormalities as diagnostic markers for PV and for risk stratifying patients with ET for disease conversion to PV or IMF.  Ref: 12682874 Semin Hematol, 2003
- When Ba F3 cells were retrovirally transduced with the  **Mpl**  type SAGs, the cells showed Tm and TPO  [ **THPO** ]  dependent growth even without IL 3.  Ref: 12646182 Biochem Biophys Res Commun, 2003
- OBJECTIVE To examine the prevalence, clinical associations, and pathogenic role of autoantibodies to c  **Mpl**   [ **MPL** ] , the thrombopoietin  [ **THPO** ]  TPO  [ **THPO** ]  receptor, in patients with systemic lupus erythematosus SLE.  Ref: 12209520 Arthritis Rheum, 2002
- After the cloning of thrombopoietin  [ **THPO** ]  c  **mpl**   [ **MPL** ]  ligand  [ **THPO** ] , Tpo  [ **THPO** ]  in 199 recombinant thrombopoietic growth factors, full length glycosylated recombinant human Tpo  [ **THPO** ]  reHuTPO and polyethylene glycol conjugated megakaryocyte growth and development factor  [ **THPO** ]  PEG reHuMGDF, have been studied in humans in a variety of clinical settings.  Ref: 12361196 Clin Appl Thromb Hemost, 2002
- Finally, we have demonstrated that upon removal of TPO  [ **THPO** ]  from the supernatant,  **Mpl**  promptly reappears on the cell surface, suggesting that a pool of intracellular  **Mpl**  can be rapidly recycled to the cell surface.  Ref: 12623841 Blood< MedlineTA>Blood, 2002
- The yeast two hybrid system has been used to study the aggregation of the intracellular domain of c  **MPL**   [ **MPL** ]  in TPO  [ **THPO** ]  signal transduction.  Ref: 12167989 Sheng Wu Hua Xue Yu Sheng Wu Wu Li Xue Bao Shanghai < MedlineTA>Sheng Wu Hua Xue Yu Sheng Wu Wu Li Xue Bao Shanghai , 2002
- In the current study, we examined the in vitro efficacy of modified SAGs containing the thrombopoietin  [ **THPO** ]  TPO  [ **THPO** ]  receptor c  **Mpl**   [ **MPL** ]  gene instead of GCR as a more potent signal generator.  Ref: 12646182 Biochem Biophys Res Commun, 2003
- A selective effect of  **Mpl**  ligand  [ **THPO** ]  on mRNA stabilization during megakaryocyte differentiation.  Ref: 12220674 FEBS Lett, 2002
- OBJECTIVE The development of megakaryocytes is regulated by thrombopoietin  [ **THPO** ]  TPO  [ **THPO** ] , which binds to the c  **mpl**   [ **MPL** ]  receptor, and by several other cytokines such as interleukin IL 6, IL 11, leukemia inhibitory factor LIF, cilliary neurotropic factor CNTF, and oncostatin OSM, which bind to gp130 protein coupled receptors.  Ref: 12135673 Exp Hematol, 2002
- Thrombopoietin  [ **THPO** ]  receptor c  **mpl**   [ **MPL** ]  is expressed on hematopoietic progenitors and cells of the megakaryocytic lineage.  Ref: 12130527 Blood, 2002
- A corollary of this finding is that TPO  [ **THPO** ]  or the c  **Mpl**   [ **MPL** ]  receptor is a candidate for involvement in the genesis of chronic myeloproliferative diseases MPD, disorders of hematopoietic stem cells.  Ref: 12682875 Semin Hematol, 2003
- We have previously shown that genetically modified marrow cells from mice can proliferate for up to a year in culture in response to CID initiated signals arising from the thrombopoietin  [ **THPO** ]  receptor  **mpl** .  Ref: 12529553 Stem Cells, 2003
- Serum TPO  [ **THPO** ]  levels in thrombocytopenic SLE patients with anti c  **Mpl**   [ **MPL** ]  antibodies were significantly elevated compared with levels in those without the antibodies P = 0.007.  Ref: 12209520 Arthritis Rheum, 2002
- This study investigates the potential role of the recombinant c  **mpl**   [ **MPL** ]  ligands recombinant human thrombopoietin  [ **THPO** ]  rhTPO and pegylated recombinant human megakaryocyte growth and development factor  [ **THPO** ]  PEG rhMGDF on the recovery of platelet counts after TBI with and without allogeneic hematopoietic stem cell transplantation HSCT in an established canine model.  Ref: 12171482 Biol Blood Marrow Transplant, 2002
- The TPO  [ **THPO** ]  receptor c  **MPL**   [ **MPL** ]  abnormalities including reduced  **MPL**  protein levels or defective TPO  [ **THPO** ]  induced signal transduction pathways are the likely mechanisms.  Ref: 12479847 Leuk Res, 2003
- Here we describe a rare case of SSc accompanied by thrombocytopenia and megakaryocytic hypoplasia, in which autoantibody against thrombopoietin  [ **THPO** ]  receptor c  **Mpl**   [ **MPL** ]  was detected.  Ref: 12794833 Arthritis Rheum, 2003
- Simultaneous measurement of serum thrombopoietin  [ **THPO** ]  and expression of megakaryocyte c  **Mpl**   [ **MPL** ]  with clinical and laboratory correlates for myelofibrosis with myeloid metaplasia.  Ref: 12068799 Eur J Haematol, 2002
- In contrast, addition of neutralizing anti c  **Mpl**   [ **MPL** ]  or anti CXCR4 Abs did not influence colony formation, indicating that this synergistic effect was independent of TPO  [ **THPO** ]  or SDF 1.  Ref: 12482498 Exp Hematol, 2002
